# A CRM1-Mediated Nuclear Export Signal Is Essential for Cytoplasmic Localization of Neurogenin 3 in Neurons

**DOI:** 10.1371/journal.pone.0055237

**Published:** 2013-01-30

**Authors:** Julia Simon-Areces, Estefania Acaz-Fonseca, Isabel Ruiz-Palmero, Luis-Miguel Garcia-Segura, Maria-Angeles Arevalo

**Affiliations:** Instituto Cajal, CSIC, Madrid, Spain; University of Iowa, United States of America

## Abstract

Neurogenin 3 (Ngn3), a proneural gene, regulates dendritogenesis and synaptogenesis in mouse hippocampal neurons. Ngn3 is transiently exported from the cell nucleus to the cytoplasm when neuronal polarity is initiated, suggesting that the nucleo-cytoplasmic transport of the protein is important for its action on neuronal development. In this study, we identified for the first time a functional nuclear export sequence (NES2; ^131^YIWALTQTLRIA^142^) in Ngn3. The green fluorescent protein (EGFP)-NES2 fusion protein was localized in the cytoplasm and its nucleo-cytoplasmic shuttling was blocked by the CRM1 specific export inhibitor leptomycin B. Mutation of a leucine residue to alanine (L135A) in the NES2 motif resulted in both cytoplasmic and nuclear localization of the EGFP-NES2 fusion protein and in the nuclear accumulation of ectopic full-length myc-Ngn3. In addition, point mutation of the leucine 135 counteracted the effects of Ngn3 on neuronal morphology and synaptic inputs indicating that the cytoplasmic localization of Ngn3 is important for neuronal development. Pharmacological perturbation of the cytoskeleton revealed that cytoplasmic Ngn3 is associated with microtubules.

## Introduction

Neurogenins (Ngn1, Ngn2 and Ngn3) are basic-helix-loop-helix (bHLH) proteins, which define a subfamily of atonal-related genes [1]–[3]. Ngn1 and Ngn2 function as proneural genes in the peripheral nervous system and thus promote both neurogenesis and notch-delta mediated lateral inhibition [4]. Ngn2 regulates neuronal subtype identity in the cerebral cortex, promoting glutamatergic cell fate [5], [6]. Ngn2 is also expressed in the progenitors that generate most dentate granule cells and has an essential role in the development of the dentate gyrus [7].

Ngn3, the third member of the neurogenin family, is expressed in the embryonic pancreas as well as in the developing central nervous system [3], [8]–[10]. Ngn3 acts as a genetic switch that specifies an endocrine cell fate in pluripotent pancreatic [11]–[13] and intestinal progenitor [14], [15]. In the nervous system Ngn3 regulates gliogenesis in the developing vertebrate spinal cord [16], neurogenesis in the retina [17] and neurogenesis and neuronal subtype specification in the hypothalamus [9]. Ngn3 is also involved in the regulation of neuronal development in the hippocampus. Overexpression of Ngn3 in cultured hippocampal neurons stimulates the outgrowth of new dendrites and induces an increase in the ratio of excitatory/inhibitory synaptic terminals [18], [19]. In addition, we have previously shown that Ngn3 is located not only in the cell nucleus but also in the perikaryon of hippocampal neurons. Furthermore, Ngn3 is transiently exported from the nucleus to the cytoplasm at critical periods of neuronal development, when neuronal polarity is established [10], suggesting that nucleo-cytoplasmic shuttling of Ngn3 is important for the function of the protein. For that reason we have explored in this study the mechanism by which Ngn3 is exported from the cell nucleus.

In eukaryotes, the cell nucleus is separated from the cytoplasm by a nuclear membrane that contains nuclear pore complexes, which allow the transport of proteins and mRNAs across the nuclear envelope [20], [21]. This process requires the proteins importin or exportin [22], [23]. Importins and exportins form ternary complexes with Ran GTPase and recognize cargo proteins that bear specific nuclear localization sequences (NLS) and nuclear export sequences (NES), respectively. The majority of shuttling proteins described to date possess a leucine-rich type of NES similar to the one originally found in the HIV-1 Rev protein. This type of NES is known to be a target recognized by the exportin CRM1 [24]. The nuclear export of Rev protein is likely conducted by binding NES to the exportin CRM1, because the shuttling of the protein is inhibited by the CRM1 inhibitor leptomycin B (LMB). The complex can then translocate to the cytoplasm, where GTP is hydrolyzed to GDP, and the NES-containing protein is released. The exportin-RanGDP complex diffuses back to the nucleus where GDP is exchanged to GTP by Ran-guanine nucleotide exchange factors. This process is energy-dependent as it consumes one GTP per cycle. Some studies indicate that nucleo-cytoplasmic shuttling is a novel regulatory mechanism of proteins, which may participate in differentiation and in inhibition of cell death [25]. In this study we have identified a functional nuclear export sequence in Ngn3. In addition, a point mutation on this sequence blocks both the nucleo-cytoplasmic transport of Ngn3 as well as the effect of Ngn3 on neuronal morphology and synaptic inputs.

## Results

### Prediction of Ngn3 Nuclear Export Signals

**Figure 1:**
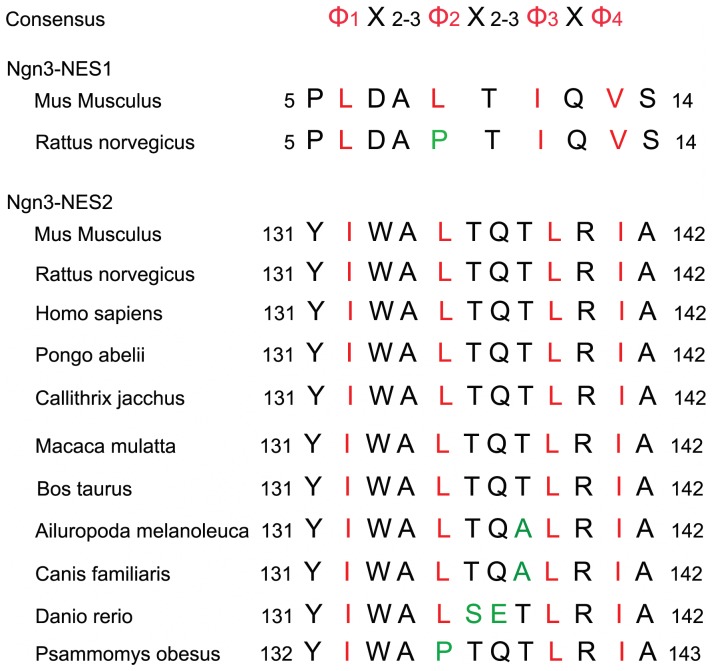
Comparison of putative nuclear export sequences (NESs) in Ngn3 protein with consensus NES motifs and Ngn3 homologues of various species. The putative NES2 sequence is well conserved in various species. In the consensus NES sequence, X indicates any amino acid and Ø indicates a hydrophobic residue, such as leucine, isoleucine, valine or methionine. Letters colored green correspond to amino acids that are not conserved in the species studied. Residues critical to NES activity are indicated by red letters.

Although it is well known that endogenous Ngn3 is localized predominantly in the cell nucleus [16], [26], we have recently shown that Ngn3 also localizes in the perikarion of developing hippocampal neurons and that the nuclear export pathway utilized by Ngn3 is CRM1-dependent [10]. This pattern of subcellular localization led us to assess whether Ngn3 has a nuclear export signal. The simplest CRM1-dependent nuclear export determinants are the so-called classic nuclear export signals (NESs). These are short peptides comprising four spaced hydrophobic residues (denoted Φ^1^–Φ^4^) and following the consensus Φ^1^-(x)_2–3_-Φ^2^-(x)_2–3_-Φ^3^-x-Φ^4^, where x is an amino acid preferentially negative charged, polar or small [27]. Therefore, we searched for NES sequences in Ngn3 and identified two potential NES motifs (NES1; ^5^PLDALTIQVS^14^ and NES2; ^131^YIWALTQTLRIA^142^) (Figure 1). NES1 is not a perfect NES consensus because it has only one amino acid between the second and third hydrophobic residues. Also the NES1 has greater acidity consistent with the lower pI predicted using the ExPASy tool (ProtParam) (3.80 for NES1 versus 8.75 for NES2). Therefore NES2 is a more robust NES motif according with the consensus sequence and it is very well conserved in a variety of species (Figure 1).

### The NES2 of Ngn3 Determines a Cytoplasmic Localization and it is CRM1 Dependent

**Figure 2:**
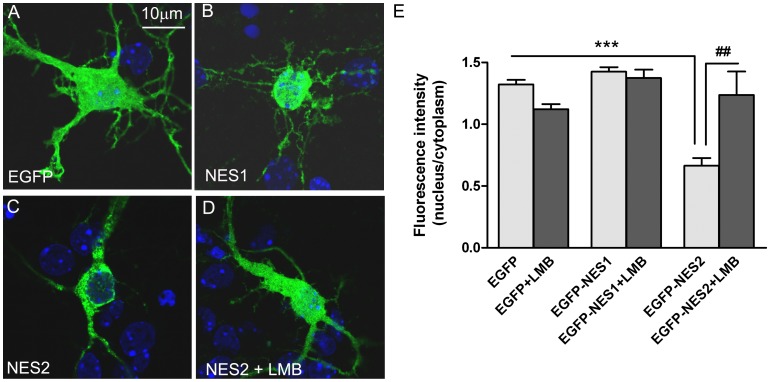
Activity of the putative nuclear export sequences (NESs) 1 and 2, with or without leptomycin B (LMB) treatment. (**A–D**) Representative single optical sections acquired by confocal microscopy of neurons expressing NES1 or NES2 fused to green fluorescent protein (EGFP). Cultured neurons were transfected at 2 DIV with expression plasmids for EGFP (A), EGFP-NES1 (B), or EGFP-NES2 (C and D) and after 16 h were treated with or without 20 nM LMB for 3 h. EGFP fluorescence was analyzed by confocal microscopy. The relative fluorescence intensity in the nucleus versus the cytoplasm was evaluated. In the absence of LMB (C) NES2 was enriched in the cytoplasm. Nuclear NES2 localization was increased in the presence of LMB (D), indicating that the signal of active nuclear exports in the Ngn3 is the NES2. (**E**) Quantification of the subcellular localization of Ngn3 in the cell nucleus versus the cytoplasm. Randomly selected fields containing cells counterstained with DAPI were digitalized and the Mean Gray Value for Ngn3 immunostaining was measured in the nuclei and cytoplasm areas using ImageJ 1.37 v software. The graphs show the mean+s.e.m. of the relative fluorescence intensity in nucleus versus cytoplasm. At least 70 cells from three experiments were counted for each experimental condition. Significance levels were determined using a Student t-test; *** p<0.001 versus EGFP expressing neurons values; ### p<0.001 of EGFP-NES2 transfection treated with LMB versus the same transfection not treated with LMB.

To establish whether these NESs can direct nuclear export, the two sequence fragments corresponding to putative NES1 or NES2 were cloned in pEGFP-C1 vector. Neuronal cultures were transfected with EGFP-NES1, EGFP-NES2 or EGFP expression vector at 2 DIV and examined by fluorescence microscopy. EGFP-NES1 was distributed in both the nucleus and the cytoplasm, like unmodified EGFP (Figure 2, A, B and E). In contrast, cells expressing EGFP-NES2 showed mainly cytoplasmic fluorescence (Figure 2, C and E). Thus, the NES2, but not NES1, of Ngn3 is sufficient to direct the exclusion of the EGFP fusion constructs from the nucleus.

Leptomycin B (LMB), a specific inhibitor of the nuclear export receptor CRM1, prevents the CRM1-NES interaction, thus inducing the accumulation of shuttling proteins into the nucleus [28]. To determine whether the nuclear export of EGFP fused to NES1 and NES2 was CRM1 dependent, we examined the effect of LMB in the subcellular localization of EGFP-NES1 and EGFP-NES2. Fluorescence microscopy demonstrated that LMB did not affect the subcellular distribution of EGFP and EGFP-NES1 (Figure 2E). In contrast, LMB increased EGFP-NES2 accumulation in the cell nucleus (Figure 2, D and E). These observations suggested that the subcellular localization of EGFP-NES2 is regulated by CRM1. Therefore, we focused our next studies on NES2.

### Mutational Analysis of NES2

**Figure 3:**
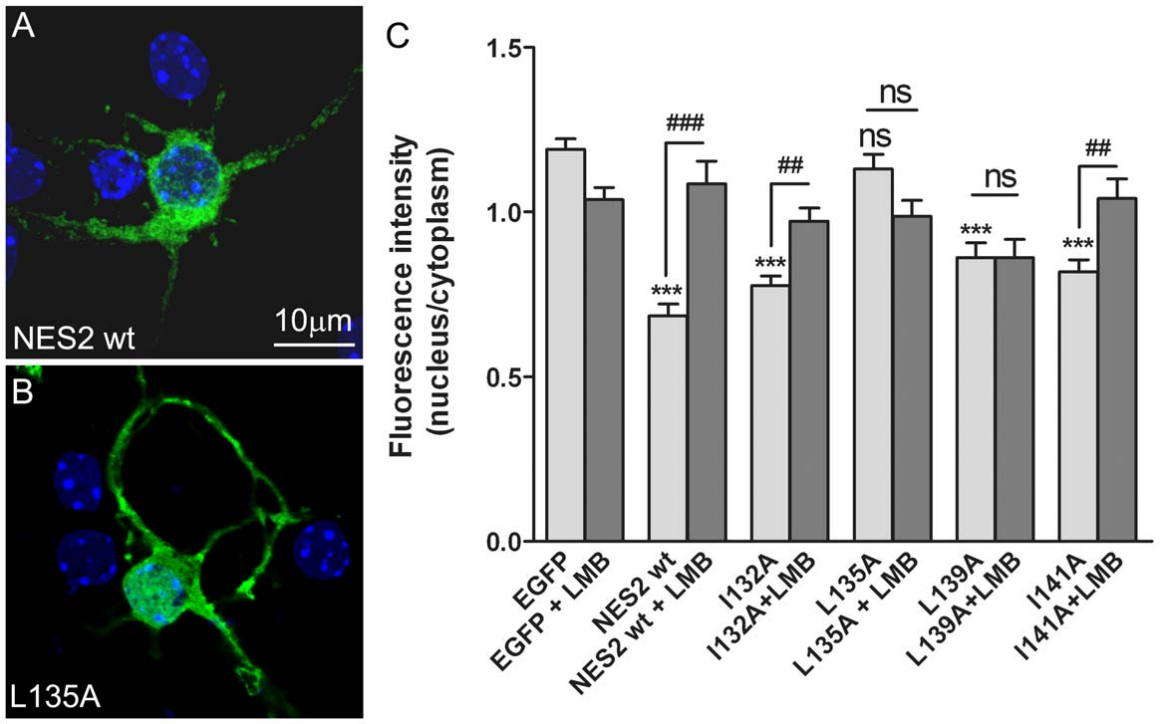
Mutational analysis of NES2. Cultured neurons were transfected at 2 DIV with expression plasmids for EGFP, EGFP-NES2, or EGFP-mutated NES2. (**A** and **B**) Representative single optical sections acquired by confocal microscopy of neurons expressing EGFP-NES2 or EGFP-NES2L135A. (**C**) Quantification of the subcellular localization of Ngn3 in the cell nucleus versus the cytoplasm. The only one mutation that was able to modify the subcellular distribution of EGFP-NES2 was L135A. In the rest of the mutations tested the location of the fusion protein continued to be mainly cytoplasmic and LMB sensitive (except for L139A that resulted LMB insensitive). The graphs show the mean+s.e.m. of the relative fluorescence intensity in nucleus versus cytoplasm. At least 70 cells from three experiments were counted for each experimental condition. Significance levels were determined using a Student’s t-test; *** p<0.001 versus EGFP expressing neurons values; ## p<0.01, ### p<0.001 versus the same transfection not treated with LMB; ns, not significant.

To explore the repercussion of NES2 amino acid sequence on nuclear export activity, we generated point mutations within NES2 in which one leucine or isoleucine residues were replaced with alanine (I132A, L135A, L139A and I141A). The NES2L135A mutant showed a significant increase in the accumulation of NES2 in the cell nucleus, compared to wild-type NES2 (Figure 3, A–C). This demonstrates that the leucine 135 is the key amino acid to export the protein out of the nucleus. The subcellular localizations of EGFP-NES2L135A and EGFP-NES2L139A were not substantially affected by LMB treatment (Figure 3 C) suggesting that both leucines are important to bind NES2 to CRM1.

### Mutation of the NES2 (L135A) Leads to Decreased Cytoplasmic Accumulation of Full-length Ngn3

**Figure 4:**
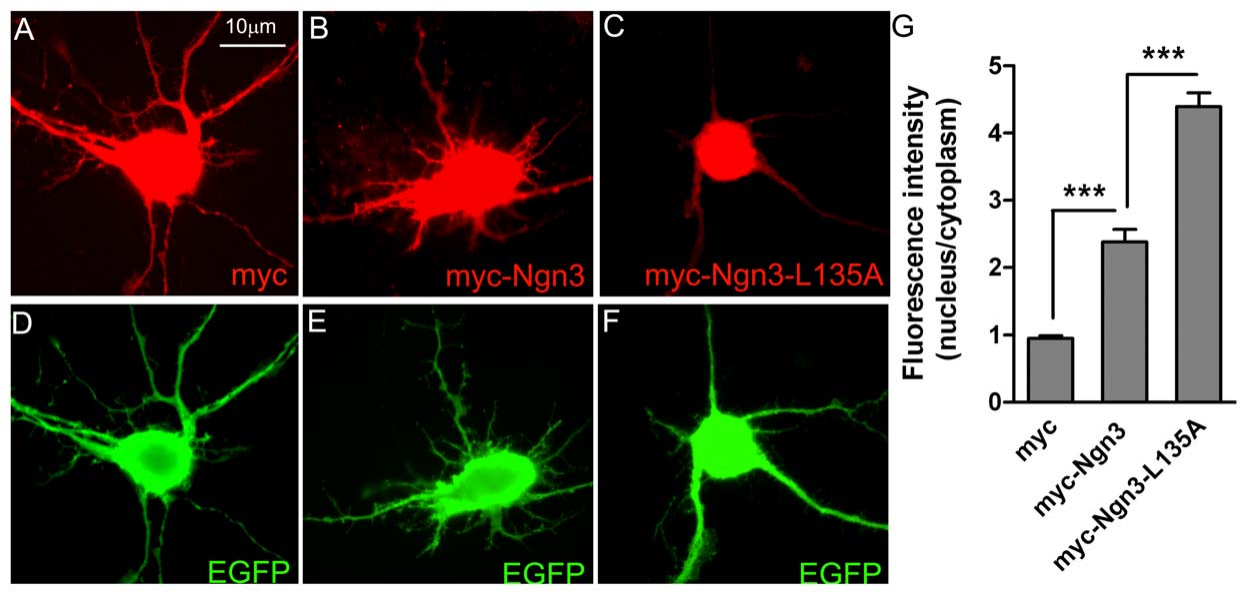
Point mutation in NES2 induces nuclear accumulation of full-length Ngn3. (**A–F**) Cultured neurons were co-transfected with constructs encoding EGFP and full-length myc-tagged wild-type Ngn3 (myc-Ngn3), Ngn3 with leucine 135 mutated to alanine (myc-Ngn3-L135A) or empty vector expressing myc-tag. After 16 h, double immunostaining was performed using an anti-myc antibody to determine subcellular localization of wild-type and mutated myc-Ngn3 (A-C) and an anti-GFP antibody to visualize neurons at full (D-F). (**G**) Quantification of the relative fluorescence in the cell nucleus versus the cytoplasm of neurons. Graphs show the results (mean+s.e.m.) of at least three experiments. Significance levels were determined using a Student’s t-test; *** p<0.001 versus values of neurons transfected with plasmid expressing myc-tag.

To investigate the role of the NES2 in exporting full-length Ngn3, we determined the effect of mutating this export sequence on Ngn3 subcelluar localization. We used site-directed mutagenesis to generate an NES2-defective version of myc-Ngn3 (myc-Ngn3-L135A) in which leucine 135 was mutated to alanine. Primary neuronal cultures were transfected with plasmids expressing myc, wild-type myc-Ngn3 or myc-Ngn3-L135A and 24 h after transfection, indirect immunostaining of myc was used to detect the proteins (Figure 4, A, B and C). Cells were cotransfected with EGFP to visualize their morphology (Figure 4, D, E and F). In cells expressing myc-tag alone the fluorescence was distributed similarly between the nucleus and the cytoplasm, including the neurites (Figure 4, A and G). In the case of wild-type myc-Ngn3, the myc fluorescence in the cytoplasm was relatively decreased in comparison to cells transfected with myc-tag alone, although significant cytoplasmic staining was detectable (Figure 4, B and G). In contrast, cells expressing myc-Ngn3-L135A showed a strong decrease in myc cytoplasmic localization (Figure 4, C and G). This was evident by the reduction in the myc labeling of the neurites, which were clearly visible with EGFP (Figure 4, C and F). Together, these results demonstrate that the NES2 reported here mediates Ngn3 nuclear export.

### Mutation of the NES2 (L135A) Impairs the Effects of Ngn3 Overexpression on the Development of Hippocampal Neurons

**Figure 5:**
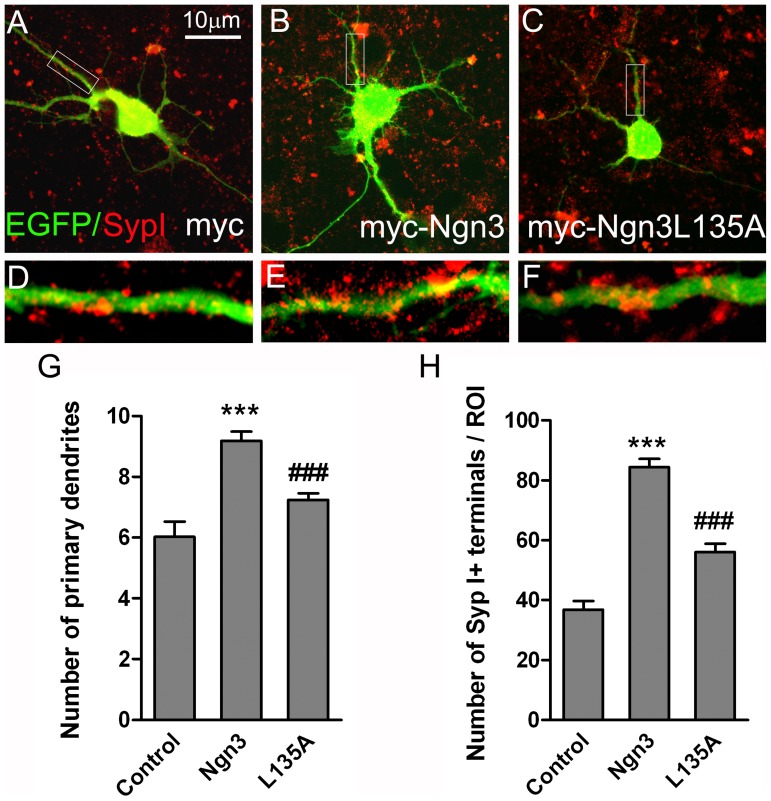
Mutation of the NES2 (L135A) counteracts the effects of Ngn3 overexpression on neuronal morphology and synaptic inputs. (**A–C**) Hippocampal neuronal cultures were co-transfected with constructs encoding EGFP and full-length myc-tagged wild-type Ngn3 (myc-Ngn3), Ngn3 with leucine 135 mutated to alanine (myc-Ngn3-L135A) or empty vector expressing myc-tag as control. After 16 h, double immunostaining was performed using an anti-GFP antibody to visualize transfected neurons and an anti-synaptophysin I antibody to determine the morphology and the total number of synapses of the transfected neurons. (**D–F**) Lower panels show the boxed regions at higher magnification (**G**) Number of primary dendrites of the transfected neurons. (**H**) Counts of synaptophysin I immunoreactive terminals in contact with a neuron within a circular region of interest (ROI) with a diameter of 100 µm and centered in the neuronal soma. Data are mean+s.e.m. and significance levels were determined using ANOVA followed by the Bonferroni post hoc test; *** p<0.001 versus control neuron values and ### p<0.001 versus myc-Ngn3 expressing neuron values.

To explore whether Ngn3 nucleo-cytoplasmic transport via CRM1 is involved in neuronal maturation, we performed a microscopic examination of dendritic processes and synaptogenesis in neuronal cultures at 4 DIV. Images in Figure 5, A–F are representative examples of neurons co-expressing EGFP and myc (Figure 5, A and D), wild type Ngn3 (Figure 5, B and E) or mutated Ngn3-L135A (Figure 5 C and F). The cultures were labeled with anti GFP and anti-synaptophysin I antibodies. Quantitative analysis showed that overexpression of Ngn3 resulted in a clear stimulation of dendrite initiation (Figure 5G) and an increase in the total number of synaptic terminals (Figure 5H). The change of leucine 135 by alanine in the Ngn3 sequence counteracts the effects of Ngn3 on the number of primary dendrites and the number of synaptic terminals. This suggests that the cytoplasmic localization of Ngn3 is important for its action on neuronal development.

### Ngn3 Co-localizes with Microtubules but not with Actin Filaments in Hippocampal Neurons

**Figure 6:**
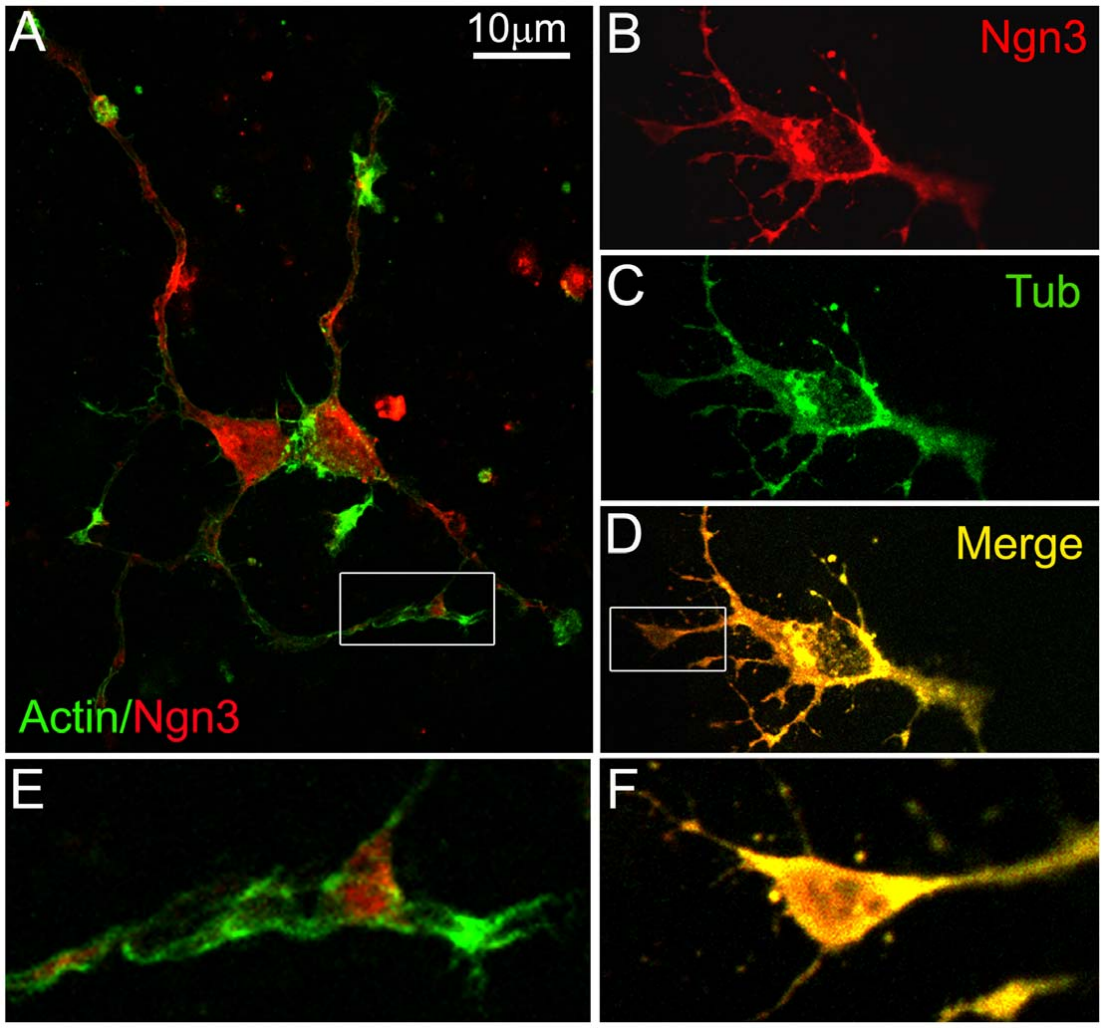
Ngn3 co-localizes with microtubules and not with actin filaments. At 3 DIV, neurons were immunostained for Ngn3 (red) and stained for actin with phalloidin (green) (panels A,E) or immunostained for Ngn3 (red) and tubulin (green) (panels B-D and F). (**A** and **D**) Representative confocal images. (**E** and **F**) Detail of growth cones. Note the association of Ngn3 with microtubules. In contrast Ngn3 was clearly not associated with actin filaments.

A study of Ngn3 sequence carried with PSORT II Prediction program showed a moderate probability of cytoskeletal localization. This cue, together with the fact that we have demonstrated its localization and impact on dendrites, lead us to analyze if Ngn3 is associated with components of the cytoskeleton. By immunocytochemical assays of neurons permeabilized in PEM buffer (80 mM PIPES, 2 mM EGTA, 1 mM MgCl_2_, pH 6.8) containing 0.05% Triton X-100 to extract soluble cytosolic proteins, we found a colocalization of Ngn3 with tubulin (Figure 6, B–D, F) but not with actin filaments (Figure 6, A, E), which were enriched in the growth cone of the cell processes (Figure 6 F).

**Figure 7:**
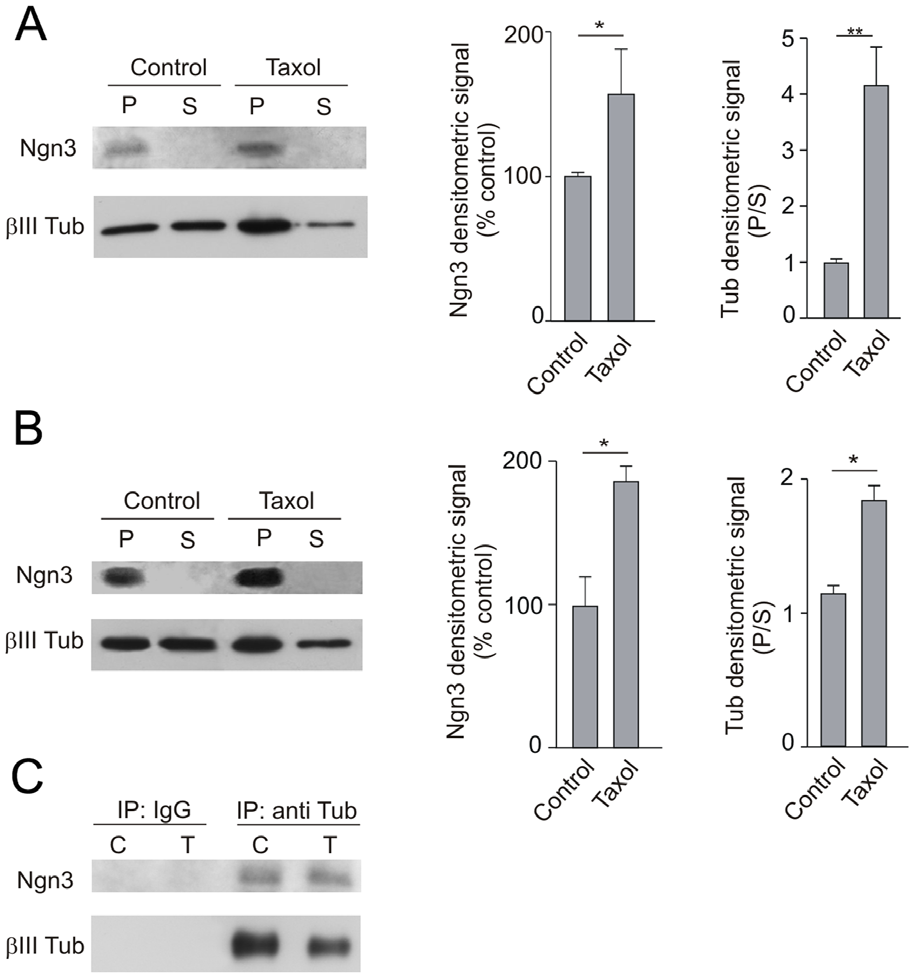
Pharmacological perturbation of the cytoskeleton suggests association of Ngn3 to tubulin and microtubules. (**A**) Cultured neurons were untreated or treated with paclitaxel (Taxol; 20 µM) for 40 minutes; then cells were lysated and centrifuged. Proteins present in the precipitate, that includes microtubules and associated proteins (P) and supernatant (S) were analyzed by Western blotting. Ngn3 is enriched in the insoluble fractions and its concentration increases as polymerized tubulin does. (**B**) Embryonic mouse brains were homogenized and a high-speed precipitate was resuspended and divided in two. Aliquots were left untreated or treated with 20 µM paclitaxel plus 1 mM GTP for 40 minutes at room temperature. Microtubular fraction sedimented by centrifugation (P) and supernatant (S) were analyzed by Western blotting. (**C**) The supernatants (S) of control (C) and paclitaxel (T) treated aliquots were immunoprecipitated (IP) with anti-βIII-tubulin antibody (or IgG control) to determine the interaction of Ngn3 with soluble tubulin. Precipitates were analyzed by Western blotting with anti-Ngn3 antibody. Graphs show the quantification of densitometry. Error bars show the mean+s.e.m. of three experiments. Significance levels were determined for the data sets connected by horizontal lines using the Student’s t-test. * p<0.05, **p<0.01.

To confirm the association between Ngn3 and microtubules, neuronal cultures were treated with paclitaxel (Taxol), a drug known to stabilize polymerized microtubules. The abundance of Ngn3 was monitored in the soluble and insoluble fractions of the cell lysates. As shown in Figure 7A, Ngn3 was enriched in the insoluble fraction. To correlate the localization of Ngn3 to the state of tubulin polymerization, the samples were also immunoblotted for βIII-tubulin. As expected, the treatment of the cultures with paclitaxel resulted in the enrichment of β-tubulin in the insoluble fraction (Figure 7A) corresponding to a stabilization of microtubules. In addition, the treatment of the cultures with paclitaxel resulted also in the enrichment of Ngn3 in the insoluble fraction (Figure 7A). Similar results were obtained in brain homogenates (Figure 7B). The treatment of brain homogenates with paclitaxel resulted in the enrichment of β-tubulin and Ngn3 in the insoluble fraction (Figure 7B). The interaction between soluble tubulin and Ngn3 was also observed by immunoprecipitation of the supernatants with anti-βIII-tubulin antibody and Western blotting of the precipitate with anti-Ngn3 antibody (Fig. 7C). Graphs in Figure 7 represent the relative amount of Ngn3 and the distribution of βIII-tubulin in the pellet and in the soluble fraction under the two experimental conditions tested in vivo and in vitro. Quantitative data are shown as the mean and standard error (s.e.m.) and statistical analyses was assessed using unpaired t-test. The levels of significance were denoted as *p<0.05, **p<0.01.

## Discussion

The scientific question we are addressing deals with the mechanism by which Ngn3 is exported from the cell nucleus during hippocampal neuronal differentiation. In this work we have demonstrated that Ngn3 possesses a functional NES that is necessary for its translocation to the cytoplasm, where Ngn3 associates with microtubules and regulates neuronal differentiation. Our conclusion is based on three observations. First, the sequence between amino acids 131 and 142 (NES2) closely fit the consensus pattern of hydrophobic residue characteristics of NESs. Second, this sequence was able to mediate nuclear export of an EGFP fusion protein and the export was inhibited by treatment with LMB. Third, point mutation of the leucine 135 in this sequence abrogated export of EGFP-NES2 and myc-Ngn3 proteins and counteracted the effects of Ngn3 on neuronal morphology and synaptic inputs. The most characterized functional role of Ngn3 is its activity as a transcription factor in endocrine cells and accordingly Ngn3 is localized in the cell nucleus of different cell types [16], [26]. Recently we have reported that Ngn3 is also localized in the cytoplasm of hippocampal neurons during specific developmental stages. Ngn3 does not remain in the cytoplasm after being synthesized; it is first transported into the cell nucleus and then is exported out again to the cytoplasm, where it is localized in neuronal processes [10]. However, the mechanism for nuclear export of Ngn3 was unknown.

### The NES Motif in the Ngn3 Sequence

Classic NESs are also referred to as leucine-rich NESs, but analyses of other export cargoes and randomization-and-selection screens showed that isoleucine, valine, methionine or phenylalanine are also permitted at the hydrophobic positions [29]–[31]. Searching for NES motif in Ngn3 sequences we identified two fragments that could function as NES according to published NES consensus sequences [32], [33]. Nevertheless only one of them, NES2, was able to direct the exclusion of the EGFP-NES2 fusion protein from the nucleus. The other one, NES1 did not exhibit nuclear export activity, probably because it do not fit as well as NES2 to the consensus sequence and also because it is a more acidic sequence than NES2. In addition, our findings suggest that the subcellular localization of EGFP-NES2 is regulated by CRM1, because the shuttling of the protein to the cytoplasm, as well as the shuttling of Ngn3, is inhibited by LMB [10]. Therefore, our data are compatible with an active nuclear export of Ngn3 mediated by an active NES located between amino acids 131 and 142.

### Key Amino Acids to Bind NES2 to CRM1

To identify the specific amino acids that are responsible to bind NES2 to CRM1, the four hydrophobic amino acids were changed by alanine and its influence in the NES2 activity was studied. The mutations I132A and I141A were well tolerated when NES2 is fused to an exogenous reporter. The mutation L135A resulted in the loss of the NES2 ability to direct the EGFP-NES2 protein to the cytoplasm and in the loss of responsiveness to LMB treatment, indicating that the leucine 135 is crucial for the NES2 binding to CRM1. Interestingly, the mutation L139A did not change NES2 nuclear export activity but provoked the disappearance of the sensibility to LMB. This suggests that such amino acid collaborates in the NES2 binding to CRM1 and enhances NES function. In the case of full length Ngn3, the point mutation L135A of the myc-Ngn3 determines virtually only nuclear protein localization. Therefore we can conclude that leucine 135 is the main amino acid for the binding of Ngn3 to exportin and that Ngn3 translocation from the cell nucleus to the cytoplasm in neurons is mediated by the exportin CRM1. This is in concordance with previous data showing that Ngn3 is retained in the cytoplasm of multiple endocrine neoplasia type 1 islet and pancreatic endocrine tumor cells [34] and that tumor size is related with the level of CRM1 expression [35].

It is of interest to note that L135A mutation had a stronger inhibitory effect on the nucleo-cytoplasmic transport of full length Ngn3 than on the nucleo-cytoplasmic transport of the NES2 fragment. The reason for this difference may be that Ngn3 has a nuclear localization site (NLS) while NES2 does not. Therefore, in the case of full-length protein the NLS promotes its translocation to the nucleus. This may also explain why in cells transfected with wild-type myc-Ngn3, the myc fluorescence in the cytoplasm was relatively decreased in comparison to cells transfected with myc-tag alone.

### Functional Consequences of Ngn3 Nucleo-cytoplasmic Transport

The finding of Ngn3 shuttling between the cytoplasm and nucleus raises the question of whether this nucleo-cytoplasmic transport has a biological function. Other authors reported a patient with two heterozygous mutations in Ngn3 and a novel subtype of permanent neonatal diabetes associated with severe malabsorptive diarrhea. One of these mutation results in the substitution of leucine for proline at position 135. They also showed that electroporation with wild-type Neurogenin 3 provokes the differentiation of delaminating and hormone expressing cells outside the normal pancreas, whereas electroporation with Ngn3L135P had no detectable effect on the chicken endoderm [36]. We have found that L135A mutation, which reduces the nucelo-cytoplasmic transport of Ngn3, causes a decrease in the number of the primary dendrites and in the number of synaptic inputs. This finding suggests that nucleo-cytoplasmic translocation of Ngn3 during neuronal development [10] is involved in the initiation and growth of dendrites.

### Ngn3 and the Cytoskeleton

The finding that the inhibition of nucleo-cytoplasmic transport of Ngn3 reduces dendritic development leads us to analyze whether Ngn3 may interact with components of the cytoskeleton. Immunocytochemical analyses showed that Ngn3 colocalizes with tubulin in cultured hippocampal neurons. The colocalization was detected in the perikaryon and the neuronal processes, including the growth cones. On the contrary, we did not detect colocalization of Ngn3 with actin filaments. In addition, Ngn3 increased in parallel with β-tubulin in the insoluble fraction of cell lysates from neuronal cultures or brain homogenates that were treated with paclitaxel to induce tubulin polymerization. Furthermore, the interaction of Ngn3 with tubulin was confirmed by immunoprecipitation experiments. These findings suggest that Ngn3 is associated with microtubules. Canonical microtubule binding motifs are present in MAP and tau proteins [37], but we have been unable to find such motifs in Ngn3. However, other proteins that lack canonical microtubule binding motifs, such as kinesins and myosins, are also able to associate to microtubules [38]–[40]. Furthermore, the interaction of Ngn3 with microtubules can be indirect and mediated by intermediate proteins. Therefore, our results suggest that the transient cytoplasmic accumulation of Ngn3 in developing neurons may mediate the actions of this molecule on the initiation of neuritogenesis.

## Materials and Methods

### Ethics Statement

CD1 mice were raised in the Cajal Institute and used to generate embryos for this study. The day of vaginal plug was defined as E0, and the mouse pups were born on E19 (P0). All procedures for handling and killing the animals used in this study were in accordance with the European Commission guidelines (86/609/CEE) and were approved by the Bioethics Committee of the “Consejo Superior de Investigaciones Científicas” (CSIC). Permit number: 28079/31A (01/18/2011).

### Antibodies

The following primary antibodies were used: chicken anti-βIII tubulin (1∶1000; Abcam, Cambridge, UK), mouse anti βIII-tubulin (Tuj1; Covance, CA; 1∶1000), mouse anti-synaptophysin I (1∶500; Progen, Heidelberg, Germany), mouse anti-myc-tag (Roche, Indianapolis, IN, USA; 1∶200), chicken anti-GFP (1∶1000; Abcam, Cambridge, UK) and mouse anti-Ngn3 (F25A1B3; 1∶2000) obtained from the NICHD Developmental Studies Hybridoma Bank (University of Iowa) with tyramide signal amplification (TSA and Cy3-TSA, PerkinElmer Life Sciences, Boston, MA) for immunostaining of Ngn3. To verify that the labelling was caused specifically by the primary antibodies, these were either omitted or replaced by similarly diluted normal serum from the same species. Peroxidase, biotin, FITC, Cy2, Cy3-conjugated secondary antibodies and peroxidase-conjugated streptavidin were from Jackson Immuno Research (West Grove, PA, USA) and Alexa 488-phalloidin (1∶1000) was from Molecular Probes (Leiden, Holland).

### Hippocampal Neuronal Cultures and Incubation Conditions

The hippocampus was dissected out from embryonic day 17 mouse embryos and dissociated to single cells after digestion with trypsin (Worthington Biochemicals, Freehold, NJ) and DNase I (Sigma-Aldrich, St. Louis, MO) [41]. Neurons were plated on 6-wells plates or glass coverslips coated with poly-L-lysine (Sigma-Aldrich) at a density of 300 neurons/mm^2^, and they were cultured in Neurobasal supplemented with B-27 and GlutaMAX I (Invitrogen, Crewe, United Kingdom). A 2–3 DIV, cultured cells were transfected and treated with either 20 nM LMB (Sigma-Aldrich) or 20 µM paclitaxel (Taxol™; Sigma-Aldrich) for the time indicated below. After the treatments, neurons were rinsed twice with PEM buffer (80 mM PIPES, 2 mM EGTA, 1 mM MgCl_2_, pH 6.8) and permeabilized with the same buffer containing 0.05% Triton X-100 (37°C, 3 min). After two rinses with PBS, cells were fixed with cold methanol (5 min, –20°C). Parallel cultures were harvested to protein analysis by Western blotting.

### Plasmid Constructs and Mutagenesis

For the construction of wild-type EGFP-NES1 and EGFP-NES2 constructs the following double-stranded oligonucleotides (Table 1) were annealed and ligated in the XhoI-EcoRI restriction sites of the expression vector pEGFP-C1 (Clontech, Palo Alto, CA).

**Table 1:** Double-stranded oligonucleotides used for the construction of wild-type EGFP-NES1 and EGFP-NES2 constructs.

NES1	5'-GAT**CTCGAG**CTCCCTTGGATGCGCTCACCATCCAAGTGTCCT **GAATTC**TGC-3'
NES1_ antisense	5'-GCA**GAATTC**AGGACACTTGGATGGTGAGCGCATCCAAGGGAG **CTCGAG**ATC-3'
NES2	5'-GAT**CTCGAG**CTTACATCTGGGCACTGACTCAGACGCTGCGCA TAGCGT**GAATTC**TGC-3'
NES2_ antisense	5'-GCA**GAATTC**ACGCTATGCGCAGCGTCTGAGTCAGTGCCCAGA TGTAAG**CTCGAG**ATC-3'

Mutations of the EGFP-NES2 expression plasmid were created using the QuikChange II site-directed mutagenesis kit protocol, according to the manufacturer's specifications (Stratagene, La Jolla, CA). All PCR-generated clones were confirmed by sequencing. Primer sequences are specified in Table 2.

**Table 2:** Primer sequences used for construction of EGFP-NES2 mutants.

I132A	5'-CTCAGATCTCGAGCTTACGCCTGGGCACTGACTCAGAC-3'
I132A_ antisense	5'-GTCTGAGTCAGTGCCCAGGCGTAAGCTCGAGATCTGAG-3'
L135A	5'-GCTTACATCTGGGCAGCGACTCAGACGCTGCG-3'
L135A_ antisense	5'-CGCAGCGTCTGAGTCGCTGCCCAGATGTAAGC-3'
L139A	5'-GGCACTGACTCAGACGGCGCGCATAGCGTGAATT-3'
L139A_ antisense	5'-AATTCACGCTATGCGCGCCGTCTGAGTCAGTGCC-3'
I141A	5'-GACTCAGACGCTGCGCGCAGCGTGAATTCTGCAG-3'
I141A_ antisense	5'-CTGCAGAATTCACGCTGCGCGCAGCGTCTGAGTC-3'

Construction of the wild-type myc-Ngn3 expression plasmid (pCS2-myc-Ngn3) has been described previously [18]. The L135A mutation of Ngn3 was created directly in the pCS2-myc-Ngn3 construct using the QuikChange II site-directed mutagenesis kit. The primers used in this case are specified in Table 3. All the plasmids were verified by DNA sequencing.

**Table 3:** Primer sequences used for construction of pCS2-myc-Ngn3-L135A.

L135A	5'-CAACTACATCTGGGCAGCGACTCAGACGCTGCGC-3'
L135A_ antisense	5'-GCGCAGCGTCTGAGTCGCTGCCCAGATGTAGTTG-3'

### Transfection

Neurons were transfected at 3 DIV using the Effectene Transfection Reagent (Qiagen GmbH, Hilden, Germany), following the manufacturer’s instructions. Cells were transfected with plasmids and after 16 h of expression time the cultures were fixed for immunostaining.

### Microtubule Association Assays

To examine Ngn3-microtubule interaction in the cultures, cells were treated with paclitaxel (Taxol; 20 µM) for 40 minutes, homogenized in PEM buffer containing 0.05% Triton X-100 and protease inhibitors (Roche Diagnostics, Mannheim, Germany) and centrifuged at 4°C for 5 minutes at 1000×g to eliminate the nucleus and membrane fractions. Supernatants were centrifuged at 4°C for 30 minutes at 100,000×g. The pellets (containing microtubules and associated proteins) were then directly depolymerized in SDS-PAGE sample buffer while soluble proteins were supplemented with 5× concentrated SDS-PAGE sample buffer. Both fractions were separated by SDS-PAGE, blotted and processed for immunodetection with anti-Ngn3 and anti-tubulin antibodies.

To examine Ngn3-microtubule interaction in vitro, embryonic mouse brains were homogenized in 10 volumes of PEM buffer containing 0.05% Triton X-100 and protease inhibitors, using 50 strokes in a glass-Teflon homogenizer. The homogenate was centrifuged at 16,000×g for 30 minutes at 4°C to eliminate the nuclear and membrane fractions and the supernatant was spun at 100,000×g for 30 minutes at 4°C. The resulting pellet was resuspended and divided in two. Aliquots were left untreated or treated with 20 µM paclitaxel (plus 1 mM GTP) for 40 minutes at RT. Aliquots were then centrifuged at 100,000×g for 30 minutes at 4°C. The pellet and supernatant fractions were then collected and analyzed by Western blotting.

### Immunoprecipitation

The supernatants from the high speed centrifugation of the brain homogeates were incubated overnight with mouse anti-βIII-tubulin antibody or with a not relevant mouse ascetic liquid at 4°C. Protein G agarose beads were then added and the incubation was maintained for 2 additional hours. The beads were washed extensively and boiled in the SDS loading buffer without reducing agent, and the precipitated proteins were detected by SDS-PAGE and Western blotting.

### Western Blotting

Proteins were resolved by SDS-PAGE and transferred onto polyvinylidene difluoride membranes (Millipore Ibérica, Madrid, Spain). The membranes were blocked in Tris-buffered saline containing 0.3% Tween 20 and 5% fat-free dry milk and incubated first with primary antibodies and then with horseradish peroxidase-conjugated secondary antibodies. Specific proteins were visualized with enhanced chemiluminescence detection reagent according to the manufacturer’s instructions (Amersham, GE Healthcare Europe GmbH, Barcelona, Spain). Densitometry and quantification of the bands were carried out using the Quantity One software (Bio-Rad, Hercules, CA).

### Image Acquisition and Analysis of Labelled Hippocampal Neurons

Images were acquired digitally using a 40× oil immersion objective and fluorescence filters. Confocal analysis was performed in a Leica (Bensheim, Germany) microscope. Photomicrographs were stored and digitally processed with Adobe Photoshop, v. 7.0 (Adobe Systems, San Jose, CA). Only minor adjustments to brightness and contrast were made. In order to quantify the Ngn3 nuclear localization signal by immunocytochemistry, randomly selected fields containing cells counterstained with DAPI were digitalized, and the Mean Gray Value for Ngn3 immunostaining in the nucleus and cytoplasm was measured using ImageJ 1.37 v software. The values were background subtracted using the average Mean Gray Value of the preparation background in each of the experimental conditions. Data were represented as relative fluorescence intensity of the cell nucleus versus the cytoplasm of each neuron. Primary dendrite number at 4 DIV (i.e., the number of dendrites emerging from the soma) and synaptic terminal counts were performed manually. A circular region of interest (ROI) with a diameter of 100 µm was projected onto the labeled neuron, its center roughly coinciding with the center of the soma. Synaptic terminals contacting the perikaryon or the dendrites were counted within the circular ROI. Dendrites broken at single or multiple points were defined as fragmented dendrites. Quantitative data are shown as the mean and standard error (mean+s. e. m.) from about 70 cells per experimental condition. Statistical analyses were performed using GraphPad Prism 5 (GraphPad Software, Inc., San Diego, CA). The one-way analysis of variance (ANOVA) was used for multiple statistical comparisons. When justified by the ANOVA analysis, differences between individual group means were analyzed by the Bonferroni post-hoc test. The Student t-test was used to compare two independent groups.
